# Endoscopic self-expandable metal stent placement before conversion therapy for advanced and recurrent gastrointestinal malignancies: A single-center retrospective study

**DOI:** 10.1097/MD.0000000000051096

**Published:** 2026-10-16

**Authors:** Qiang Liu, Rong Yu, Jisheng Wang, Xiaoqin Zong, Ming Bai

**Affiliations:** a Department of Gastrointestinal Surgery, Gansu Wuwei Academy of Medical Science, Wuwei, Gansu, China; b Personnel Section, Gansu Wuwei Academy of Medical Science, Wuwei, Gansu, China; c Wuwei Municipal Health Bureau, Wuwei, Gansu, China.

**Keywords:** conversion therapy, endoscopy, gastrointestinal malignancy, metal stent

## Abstract

Endoscopic self-expandable metal stent (SEMS) placement is an established palliative treatment for malignant gastrointestinal (GI) obstruction, but data on its use in patients eligible for subsequent conversion therapy remain limited. We describe our single-center experience with SEMS placement in advanced and recurrent GI malignancies and report the proportions of patients who subsequently completed antitumor therapy and underwent R0 resection. We retrospectively reviewed 148 consecutive patients with advanced or recurrent GI malignancies who underwent SEMS placement for malignant obstruction or fistula at a single center between January 2019 and December 2022. Technical success, clinical success, fistula closure, adverse events, subsequent antitumor therapy, R0 resection, and overall survival were assessed. All analyses were descriptive and exploratory; no adjustment for confounding was performed. Gastrointestinal SEMSs were successfully placed in 146 patients (98.6%). The overall obstruction-relief rate was 95.7%, and the overall fistula-closure rate was 80%. No patient in this cohort required rescue gastrostomy or colostomy because of failed stent therapy during the study period. A total of 132 patients (94.3%) completed antitumor treatment after endoscopic metal stent placement (EMSP). R0 resection was performed in 8.89% of patients with esophageal malignancies, 10.10% of those with gastric malignancies, and 17.9% of those with colorectal malignancies. Median survival time (MST) was generally longer among patients who underwent conversion surgery than among those who did not. Comorbidities, anemia, and SEMS-related adverse events were also analyzed as factors associated with conversion therapy after SEMS placement; the respective *P* values were .039, <.001, and .012. In this retrospective single-center series, SEMS placement was technically feasible and relieved obstruction or closed fistulas in most patients with advanced or recurrent GI malignancies; a subset subsequently completed antitumor therapy and underwent R0 resection. These data support feasibility and demonstrate associations but cannot establish a survival benefit; prospective comparative studies are required.

## 1. Introduction

Gastrointestinal (GI) cancers impose a disproportionate global burden: the 5 major GI cancers – esophageal, gastric, colorectal, liver, and pancreatic – together account for approximately 1-quarter of global cancer incidence and approximately one-third of all cancer-related deaths.^[1,2]^ GI tumors are often diagnosed at an incurable, advanced stage and are associated with a poor prognosis. Conversion therapy for GI tumors has therefore received increasing attention. It is defined as surgery intended to achieve no residual tumor (R0) resection after chemotherapy or radiotherapy for tumors initially considered unresectable or marginally resectable for technical and/or oncological reasons.^[3]^ However, many patients with advanced GI tumors cannot tolerate further antitumor therapy because of GI stenosis or fistulas. Since GI stents were first used in 1885, their design and applications have evolved in response to clinical needs and technological advances. The introduction of covered metal stents in 1994 helped address tumor ingrowth and embedding of the stent mesh. Nitinol stents were subsequently introduced; their shape-memory and superelastic properties allow them to maintain an appropriate configuration, thereby reducing mucosal injury and facilitating safer removal.^[4,5]^ These advances have expanded treatment options for GI malignancies. Recent European Society of Gastrointestinal Endoscopy (ESGE) guidelines and several clinical studies recommend self-expandable metal stents (SEMSs) for palliation of malignant intestinal obstruction and stenting as a bridge to surgery (BTS).^[6-8]^ Nevertheless, few studies have examined SEMS placement as a bridge to conversion surgery.

We therefore reviewed our institutional experience with SEMS placement in 148 patients with advanced or recurrent gastrointestinal malignancies treated between January 2019 and December 2022, with 4 separate objectives: to quantify technical success of stent placement across anatomical sites; to quantify clinical success, defined as relief of obstruction or closure of fistula according to site-specific criteria; to determine the proportion of patients who subsequently completed systemic antitumor therapy and the proportion who underwent R0 resection; and to describe overall survival according to whether conversion surgery was performed. Given the retrospective, single-center, uncontrolled design, all 4 objectives are addressed descriptively.

## 2. Materials and methods

### 2.1. Participants

This was a single-center retrospective observational cohort study. The primary objective was descriptive: to quantify the technical success, clinical success, and adverse events of endoscopic SEMS placement in patients with advanced or recurrent GI malignancies. Secondary analyses comparing survival according to conversion status and examining characteristics associated with proceeding to conversion therapy were exploratory and hypothesis-generating. No control group was assembled, no adjustment for confounding or multiplicity was performed, and no causal inference was intended. The study is reported in accordance with the Strengthening the Reporting of Observational Studies in Epidemiology (STROBE) statement for observational studies.^[9]^

The inclusion criteria were as follows: an advanced or recurrent malignant GI tumor confirmed by histopathological or imaging examination; a tumor judged unresectable after multidisciplinary team (MDT) discussion; obstruction or a GI fistula before conversion therapy; and follow-up from the date of stent placement until death from any cause or the data cutoff in December 2022, whichever occurred first. A minimum follow-up of 30 months was required for inclusion in the survival analysis. Patients who underwent stenting within 30 months of the cutoff were included in the descriptive analyses of technical and clinical success but were excluded from the survival analysis.

The exclusion criteria were as follows: mild symptoms and a stenosis traversable with an adult endoscope; clinical evidence of perforation and peritonitis; or clinical evidence of obstruction at multiple sites.

### 2.2. GI stenting procedure

#### 2.2.1. Preoperative examination and anesthesia assessment

Before metal stent placement, lesions at the relevant GI anatomical sites were evaluated by imaging. Representative lesions included esophageal stenosis (Fig. 1A), esophagotracheal fistula (Fig. 1B), postoperative recurrence of gastric cancer with a fistula (Fig. 1C), and colorectal obstruction (Fig. 1D).

**Figure 1.**
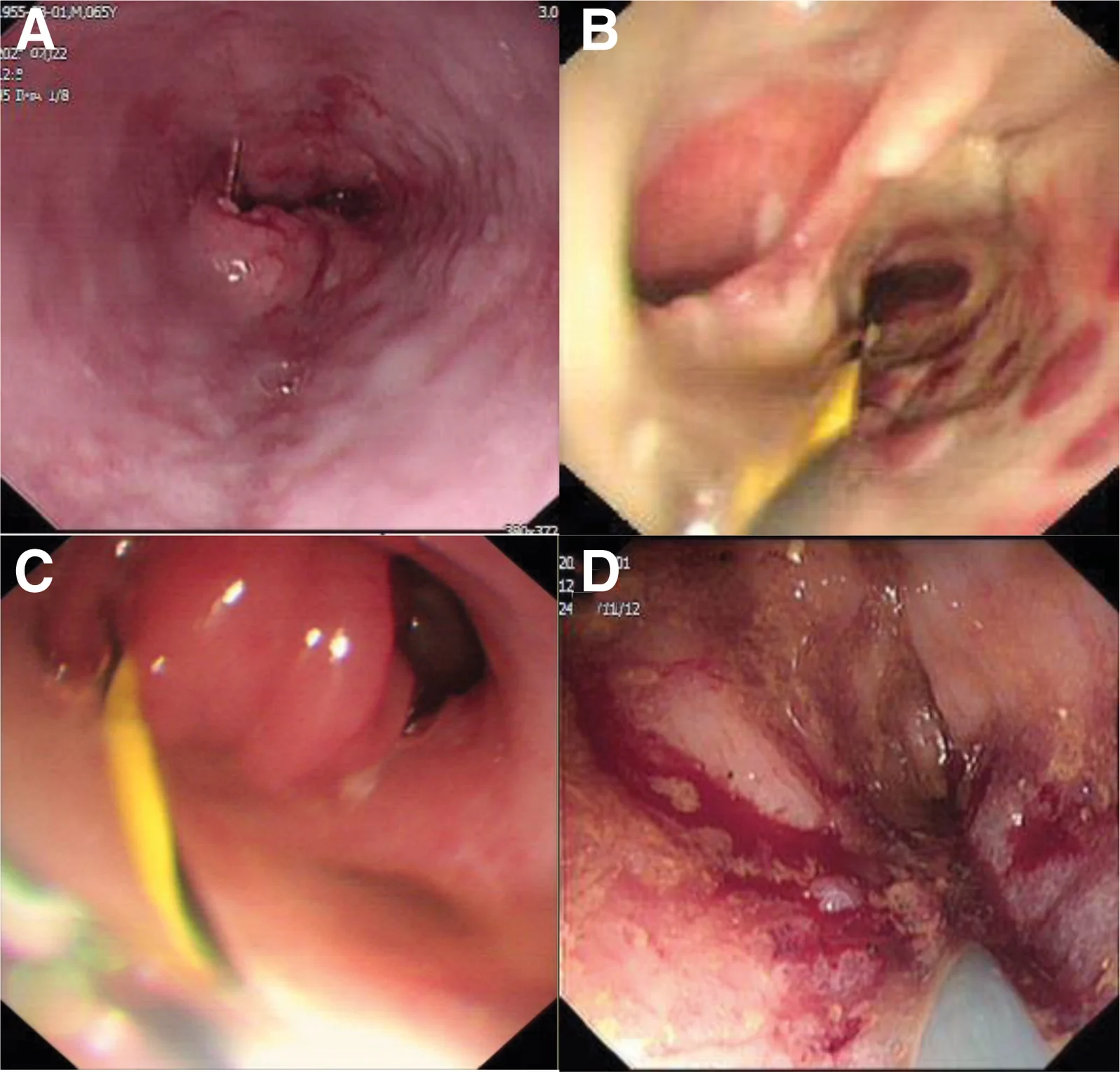
Representative endoscopic findings before self-expandable metal stent placement. (A) Malignant esophageal stenosis. (B) Esophagotracheal fistula following radiotherapy. (C) Fistula associated with recurrent gastric cancer after radical resection. (D) Malignant colorectal obstruction.

Each patient’s American Society of Anesthesiologists (ASA) physical status was assessed on the basis of the medical history, comorbidities, laboratory findings, and ancillary examinations. Patients were informed about the purpose of stent placement, the results of the anesthetic assessment, intraoperative risks, and precautions to follow after stent placement.

#### 2.2.2. Procedural details

Five minutes before endoscopic stent placement, dyclonine hydrochloride gel or lidocaine mucilage was administered for local pharyngeal anesthesia, and the endoscope was introduced through the oropharynx. Depending on the procedural requirements, the patient was placed in the left lateral, supine, or right lateral position to optimize lesion exposure and facilitate the procedure.

A gastroscope (Olympus GIF-H290), ultrathin gastroscope (Olympus GIF-XP260N), duodenoscope (Olympus TJF-260V), or colonoscope (Olympus CF-HQ290I) was selected and advanced to the lesion (stenosis or fistula) under direct endoscopic visualization. Stents were selected according to the lesion site as follows: partially or fully covered SEMSs for esophageal lesions; partially covered SEMSs or uncovered metal stents for gastric outlet obstruction; and uncovered metal stents when duodenal obstruction involved the ampulla of Vater or when the colonic lesion was <5 cm long. The procedures for esophageal, pyloric, and colonic SEMS placement are shown in Figure 2A–E, F–J, and K–O, respectively.

**Figure 2.**
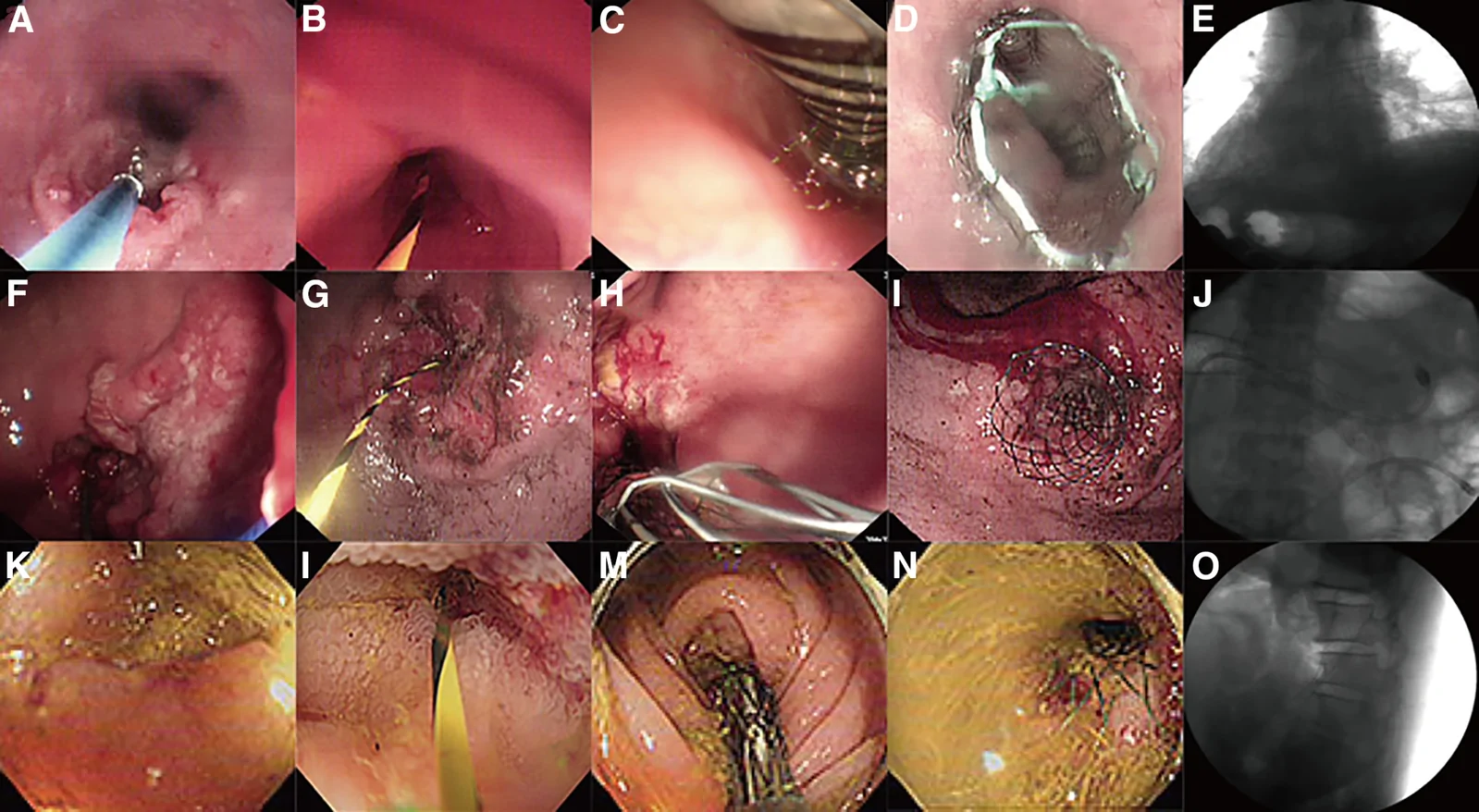
Sequential endoscopic and fluoroscopic images of self-expandable metal stent placement at different gastrointestinal sites. (A–E) Esophageal SEMS placement; (F–J) pyloric SEMS placement; and (K–O) colonic SEMS placement. SEMS = self-expandable metal stent.

All procedures were performed by 2 senior endoscopists (each with more than 10 years of therapeutic endoscopy experience and an annual volume of more than 500 stent placements) within a single gastrointestinal endoscopy unit. Fluoroscopic guidance was used in all cases.

#### 2.2.3. Postoperative therapy

Basic vital signs were closely monitored after the procedure. Patients remained nil per os (NPO) for 3 to 4 hours and then drank an appropriate amount of warm water to promote full stent expansion. Patients with fistulas in the upper GI tract received an endoscopic nasojejunal feeding tube as a transitional nutritional measure. One week after the procedure, radiography was performed to assess stent position and expansion.

After satisfactory SEMS placement had been confirmed, patients were evaluated for their ability to tolerate further antitumor therapy. Subsequent R0 resection was regarded as successful conversion therapy. Gastroscopy or colonoscopy was performed periodically during subsequent systemic management.

### 2.3. Observation outcomes

Technical success was defined as deployment of the stent across the full length of the stricture or fistula in the intended position within a single session, confirmed by endoscopy and by fluoroscopy or by plain radiography. Clinical success (relief of obstruction) was assessed in patients stented for obstruction and was defined separately by anatomical site. For esophageal and cardia lesions, it was defined as an improvement of at least 1 Stooler dysphagia grade between the pre-procedural assessment and day 1 to 3 after stenting. For gastric-outlet, duodenal, and colorectal lesions, it was defined as resolution or clinically meaningful reduction of obstructive symptoms (abdominal distension, pain, vomiting, or difficulty in defecation) recorded in the medical record within 3 days of stenting. Fistula closure was defined as absence of contrast extravasation on a water-soluble contrast study, absence of a visible fistula orifice at endoscopy, or both. Fistula closure was assessed only in the patients stented for a fistula and was not pooled with obstruction outcomes. Oncological outcomes were process and efficacy measures downstream of stenting and are reported separately from stent performance: administration of at least 1 cycle of systemic antitumor therapy after stenting; conversion surgery; and overall survival, measured from the date of stent placement. Adverse events were classified and graded according to the American Society for Gastrointestinal Endoscopy (ASGE) lexicon.^[10]^

Conversion surgery was defined, following the framework of Yoshida et al,^[11]^ as surgical resection undertaken with curative intent after systemic antitumor therapy in a patient whose disease had been judged unresectable at the multidisciplinary team assessment preceding stent placement. Successful conversion therapy was defined as such surgery achieving R0 resection, that is, complete macroscopic resection with histologically negative margins on pathological examination of the specimen. Patients who underwent resection with microscopic residual tumor (R1) or macroscopic residual tumor (R2) margins were classified as not having achieved successful conversion therapy.

### 2.4. Statistical analysis

Data were processed and analyzed using Statistical Package for the Social Sciences (SPSS) version 26.0. Quantitative data were summarized as the mean ± standard deviation and compared using the *t* test. Categorical data were summarized as frequencies and percentages and compared using the chi-square test. An improvement of at least 1 grade in the Stooler dysphagia score within 1 to 3 days after stent placement was used to assess clinical success. Statistical significance was defined as *P* < .05. Survival curves were generated using the Kaplan–Meier method, and median survival time (MST) was calculated.

## 3. Results

### 3.1. Patients

Between January 2019 and December 2022, 148 consecutive patients with advanced or recurrent gastrointestinal malignancies underwent SEMS placement for malignant obstruction or fistula. The indication was disease progression causing stenosis in 125 patients, recurrent obstruction after radical resection in 17, recurrent fistula after radical resection in 4, and esophagotracheal fistula following radiotherapy in 2. The primary tumor was esophageal in 45 patients, gastric in 69, duodenal in 6 and colorectal in 28. Baseline characteristics are shown in Table 1. Stents were placed at 6 positions: esophagus (n = 45), cardia (n = 39), pylorus (n = 30), duodenum (n = 6), colon (n = 21), and rectum (n = 7). Of the 148 patients, 142 were stented for obstruction and 6 for a fistula.

**Table 1:** Baseline characteristics of patients.

Items	Esophageal cancer (n = 45)	Gastric cancer (n = 69)	Duodenal cancer (n = 6)	Colorectal cancer (n = 28)
Sex (%)				
Male	33 (73)	47 (68)	4 (67)	19 (68)
Female	12 (27)	22 (32)	2 (33)	9 (32)
Age				
Median (IQR)	63 (13)	57 (15)	60 (9)	66 (18)
Mean (±SD)	67 ± 10.18	62 ± 11.13	64 ± 8.94	57 ± 14.43
ASA class (%)				
I	7 (15.56)	13 (18.84)	2 (33.33)	12 (42.86)
II	29 (64.44)	34 (49.28)	3 (50.0)	9 (32.14)
III	9 (20.0)	18 (26.09)	1 (16.67)	6 (21.43)
IV	0 (0)	4 (5.79)	0 (0)	1 (3.57)
TNM stage (%)				
cT				
2	3 (6.67)	4 (5.80)	1 (16.67)	2 (7.14)
3	26 (57.78)	42 (60.87)	4 (66.67)	14 (50.0)
4	16 (35.56)	23 (33.33)	1 (16.67)	12 (42.86)
cN				
(-)	9 (20.0)	3 (4.35)	2 (33.33)	7 (25.0)
(+)	36 (80.0)	66 (95.65)	4 (66.67)	21 (75.0)
M				
0	32 (71.11)	50 (72.46)	5 (83.33)	20 (71.43)
1	13 (28.89)	19 (27.54)	1 (16.67)	8 (28.57)
Previous antitumor therapy (%)				
Surgical	6 (13)	23 (33)	1 (17)	9 (32)
Non-surgical	39 (87)	46 (67)	5 (83)	19 (68)
Comorbidities (%)				
Yes	27 (60)	35 (51)	1 (17)	10 (36)
No	18 (40)	34 (49)	5 (83)	18 (64)

ASA = American Society of Anesthesiologists, IQR = interquartile range, SD = standard deviation, TNM = tumor–node–metastasis.

### 3.2. Technical success

Technical success was achieved in 146 of 148 patients (98.6%). Both failures occurred in patients with gastric malignancy. In one, a pyloric stent could not be placed because an advanced tumor had produced a stenosis that could not be dilated sufficiently to admit the delivery system. In the other, a cardiac stent could not be seated because the diameter of a post-resection fistula exceeded 1.5 cm. Technical success by stent position was 45 of 45 esophageal, 38 of 39 cardiac, 29 of 30 pyloric, 6 of 6 duodenal, 21 of 21 colonic, and 7 of 7 rectal placements. Placement was achieved in a single session in 140 of the 146 technically successful cases (95.9%). Six patients required a second placement, in each case because of a stent-related adverse event occurring after initially satisfactory deployment: stent migration in 3, stent detachment in 1, perforation in 1 and recurrent stenosis in 1.

### 3.3. Clinical success: relief of obstruction

Clinical success was assessed in the 141 patients successfully stented for obstruction. Dysphagia was graded before and after stenting using the Stooler dysphagia score in the 42 patients with esophageal and 36 with cardiac stents placed for obstruction. Dysphagia improved by at least 1 grade in 40 of 42 patients with esophageal stents (95.2%) and 35 of 36 with cardiac stents (97.2%). Grade distributions before and after placement are shown in Table 2 (*P* < .001 in both groups). Obstructive symptoms were relieved with resumption of oral intake in 27 of 29 patients with pyloric stents (93.1%) and 6 of 6 with duodenal stents (100%). Obstructive symptoms resolved with passage of flatus or stool, without further intervention, in 27 of 28 patients (96.4%). Across all sites, obstruction was relieved in 135 of 141 patients (95.7%). The overall clinical efficacy of metal stents for the treatment of GI malignancies is shown in Figure 3A.

**Table 2:** Comparison of dysphagia before and after SEMS placement.

Items	Stooler classification					
	Grade 0	Grade 1	Grade 2	Grade 3	Grade 4	*P* value
Esophageal SEMS implantation						<.001
Before	0	4	14	16	8	
After	17	8	10	5	2	
Cardiac SEMS implantation						<.001
Before	0	4	17	11	4	
After	17	10	7	1	1	

SEMS = self-expandable metal stent.

**Figure 3.**
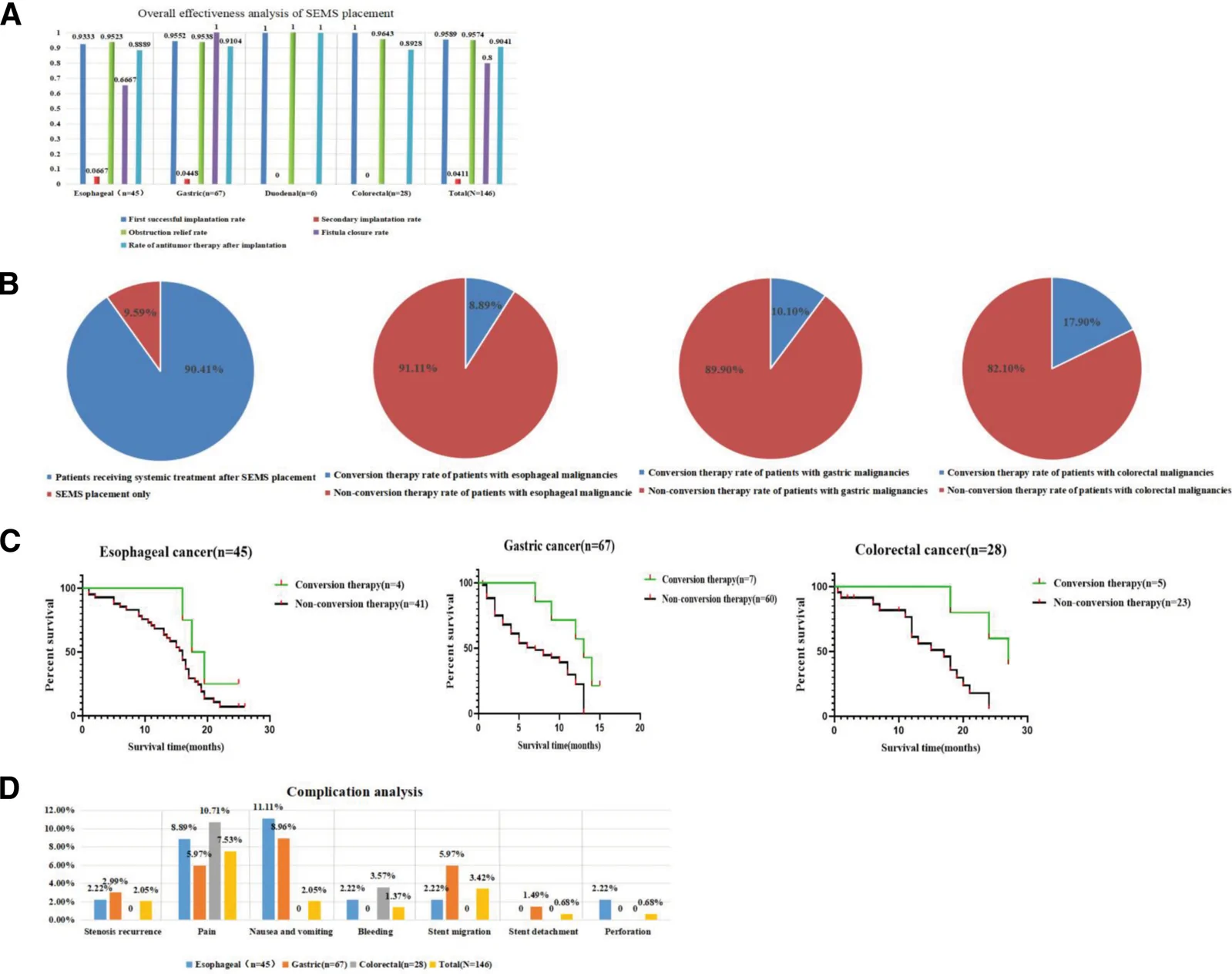
Feasibility analysis of SEMS implantation as a bridge to conversion therapy. (A) The overall clinical efficacy of metal stents for the treatment of GI malignancies. (B) Proportion of patients receiving systemic antitumor therapy after stent placement (left) and site-specific proportions of patients who did or did not undergo conversion surgery achieving R0 resection (three charts on the right). (C) Kaplan–Meier overall survival curves comparing patients who underwent conversion surgery with those who did not, separately for esophageal, gastric, and colorectal malignancies. (D) Adverse events analysis. GI = gastrointestinal, SEMS = self-expandable metal stent.

### 3.4. Fistula closure

Six patients underwent stenting for a GI fistula: 4 for recurrent fistulas after radical resection and 2 for esophagotracheal fistulas after radiotherapy. By stent position, 3 were esophageal and 3 were cardiac. Placement was technically successful in 5 patients. Fistula closure – defined as the absence of contrast extravasation on a water-soluble contrast study, the absence of a visible fistula orifice at endoscopy, or both – was achieved in 4 of the 5 successfully stented patients: 2 of 3 with esophageal fistulas and two of two with gastric fistulas. Patients with upper GI fistulas received nasojejunal feeding as a transitional measure until closure was confirmed.

### 3.5. Oncological outcomes

#### 3.5.1. Systemic antitumor therapy after stenting

Of the 146 patients in whom stent placement was technically successful, 6 – all with duodenal malignancies – declined further antitumor treatment, leaving 140 patients evaluable for oncological outcomes. The two patients in whom placement failed also received no further antitumor therapy. Of these 140 evaluable patients, 132 (94.3%) received systemic antitumor therapy after stent placement, including photodynamic therapy combined with immunotherapy, targeted therapy, or chemotherapy, as well as systemic monotherapy. The remaining 8 patients received nutritional support alone.

#### 3.5.2. Conversion surgery

Conversion surgery achieving R0 resection was ultimately performed in 16 of the 140 evaluable patients (11.4%): 4 of 45 with esophageal malignancies (8.9%), 7 of 67 with gastric malignancies (10.4%), and 5 of 28 with colorectal malignancies (17.9%; Fig. 3B).

#### 3.5.3. Overall survival

Among patients who successfully underwent conversion therapy, the MSTs for those with esophageal, gastric, and colorectal malignancies were 18.5, 13, and 27 months, respectively. The corresponding MSTs among patients who did not undergo conversion therapy were 16, 7, and 17 months, respectively (Fig. 3C).

### 3.6. Characteristics associated with conversion surgery

Among the 140 evaluable patients, the presence of comorbidity (*P* = .039), the severity of anemia (*P* < .001), and the occurrence of a stent-related adverse event (*P* = .012) were associated with whether conversion surgery was subsequently performed, whereas age, sex, tumor–node–metastasis (TNM) stage, and hypoproteinemia were not (Table 3). No patient who experienced a stent-related adverse event underwent conversion surgery (0 of 35).

**Table 3:** Correlation between conversion therapy after SEMS implantation and clinical characteristics of patients with GI malignancies.

Characteristics	Conversion therapy		*P* value
	No	Yes	
Age			.065
<60	53	3	
≥60	71	13	
Sex			.256
Male	88	9	
Female	36	7	
TNM stage			.87
II	14	2	
III	71	10	
IV	39	4	
Comorbidities			.039
Yes	65	4	
No	59	12	
Anaemia			<.001
No	9	11	
Mild	59	3	
Moderate	44	2	
Severe	12	0	
Hypoproteinemia			.828
Yes	50	6	
No	74	10	
SEMS adverse events			.012
Yes	35	0	
No	89	16	

GI = gastrointestinal, SEMS = self-expandable metal stent.

### 3.7. Adverse events

Adverse events occurred in 34 of the 146 successfully stented patients (23.3%): 13 of 45 with esophageal stents (28.9%), 17 of 67 with gastric stents (25.4%), 4 of 28 with colorectal stents (14.3%). Graded according to the ASGE lexicon, events comprised mild chest or abdominal pain in 11 patients (7.5%), self-limiting within 1 to 5 days; nausea and vomiting in 11 (7.5%), relieved by antiemetics, acupuncture, or short-term fasting; stent migration in 5 (3.4%), managed by second placement in 3 and endoscopic repositioning in 2; recurrent stenosis in 3 (2.1%), managed by a second stent in 1 and photodynamic therapy in 2; gastrointestinal bleeding in 2 (1.4%), low-volume and responsive to acid suppression; stent detachment in 1 (0.7%), requiring second placement; and perforation in 1 (0.7%), managed by second stent placement. Adverse events by anatomical site are shown in Figure 3D.

## 4. Discussion

Endoscopic SEMS placement was effective for the management of obstruction or fistula in patients with GI malignancies. During the 4-year study period, 146 of 148 patients with GI malignancies at our center underwent successful endoscopic SEMS placement under fluoroscopic guidance. The technical success, obstruction-relief, fistula-closure, and adverse-event rates were 98.6%, 95.7%, 80%, and 23.3%, respectively. The observed technical and clinical success rates were within and at the upper end of the ranges reported in previous series, although differences in case mix preclude direct comparisons.^[12-16]^ SEMS placement relieved stenosis or closed fistulas in most patients and was followed by systemic antitumor therapy in the majority. These observations are consistent with a potential facilitating role for SEMS placement, although the uncontrolled design does not permit this hypothesis to be tested.

The principal bridging options are SEMS placement and diverting stoma (DS). A disadvantage of emergency ostomy is that the stoma reportedly becomes permanent in approximately two-thirds of patients.^[17]^ Nevertheless, DS is preferred because of its safety profile in patients with acute angulated obstruction and marked proximal bowel distension.^[18]^ Given its minimally invasive nature, SEMS placement may be an alternative to DS in appropriately selected patients. In patients with fistulas, SEMS decompression may also reduce adverse events and facilitate tumor staging and the identification of synchronous lesions by imaging and preoperative endoscopy.

In this study, R0 resection was performed in 8.89% of patients with esophageal malignancies, 10.1% of those with gastric malignancies, and 17.9% of those with colorectal malignancies. These rates were lower than the 23.9% reported in a previous clinical study of SEMS placement as a bridge to conversion surgery for GI malignancies.^[19]^ We also found that comorbidities, anemia, and SEMS-related adverse events were associated with the likelihood of successful conversion therapy. These associations may help identify patients less likely to proceed to conversion surgery, but they were derived from univariable analyses with few events and require prospective confirmation. Among patients with gastric cancer, MST was significantly longer after conversion surgery than without conversion surgery (13 vs 7 months, *P* = .0345). Similarly, among patients with colorectal cancer, MST was significantly longer with conversion therapy than without it (27 vs 17 months, *P* = .0246). Among patients with esophageal cancer, however, MST did not differ significantly between those who underwent conversion surgery and those who did not (18.5 vs 16 months, *P* = .1881).

Among the 146 patients who underwent successful SEMS placement, 34 experienced adverse events. The most common were pain, nausea, and vomiting. Stent migration, instability, and detachment may be related to the placement site, stent expansion, and GI motility. Several considerations may help prevent or manage these events: for simple anastomotic stenosis, a short 5 to 7-cm stent should be selected, whereas a longer stent is appropriate for long lesions or suspected anastomotic tumor recurrence and should generally extend 1.5 to 2 cm beyond both ends of the lesion; a small-diameter stent may migrate easily, whereas an excessively large diameter may damage the GI wall and cause bleeding; and placement in the upper esophagus may injure the GI wall or induce repeated tissue repair and subsequent restenosis and should therefore be undertaken with care.

This study has several important limitations. First, it was a retrospective, single-center series without a control group; patients were not randomized to stenting, and comparisons with diverting stoma, surgical bypass, or best supportive care were not possible. Second, the cohort was heterogeneous, encompassing esophageal, cardiac, gastric-outlet, duodenal, colonic, and rectal lesions, which differ in natural history, stent design, technical difficulty, and the feasibility of subsequent resection; pooling these lesions may obscure site-specific effects. Third, several subgroups were small – 6 patients with duodenal malignancies, 5 patients successfully stented for fistulas, and 16 conversion-surgery events in total – so the corresponding proportions have wide confidence intervals, and the site-specific survival comparisons were markedly underpowered. Fourth, analyses of factors associated with conversion therapy were univariable and unadjusted. With only 16 conversion events, a multivariable model could accommodate at most 1 or 2 covariates before overfitting under the conventional requirement of approximately 10 events per variable^[20,21]^; we therefore present these associations as exploratory rather than as independent predictors, and no correction for multiple testing was applied. Fifth, the decision to proceed to conversion surgery was made by an MDT on clinical grounds; consequently, patients who underwent surgery were systematically fitter and had more chemosensitive disease, and residual confounding by indication cannot be excluded. Sixth, outcome ascertainment relied on routinely recorded clinical documentation rather than protocol-mandated assessment, and symptom relief at nonesophageal sites was not graded using a validated instrument. Finally, as a single-center experience from a high-volume unit, these results may not be generalizable to centers with different procedural expertise or referral patterns.

## 5. Conclusions

In this retrospective single-center series, SEMS placement was technically feasible, relieved obstruction or closed fistulas in most patients, and had an acceptable adverse-event profile. A subset of patients subsequently completed systemic antitumor therapy and underwent R0 resection. Whether SEMS placement itself contributes to improved survival cannot be determined from these data and requires prospective comparative evaluation.

## Author contributions

**Conceptualization:** Qiang Liu.

**Data curation:** Qiang Liu, Rong Yu, Xiaoqin Zong.

**Formal analysis:** Qiang Liu, Rong Yu, Jisheng Wang.

**Funding acquisition:** Qiang Liu, Rong Yu.

**Investigation:** Qiang Liu, Jisheng Wang, Xiaoqin Zong.

**Methodology:** Ming Bai.

**Project administration:** Rong Yu, Xiaoqin Zong.

**Resources:** Jisheng Wang, Ming Bai.

**Software:** Qiang Liu, Jisheng Wang, Ming Bai.

**Supervision:** Rong Yu, Jisheng Wang, Xiaoqin Zong, Ming Bai.

**Writing – original draft:** Qiang Liu.

**Writing – review & editing:** Ming Bai.
